# Genome sequencing and implications for rare disorders

**DOI:** 10.1186/s13023-019-1127-0

**Published:** 2019-06-24

**Authors:** Jennifer E. Posey

**Affiliations:** Department of Molecular & Human Genetics, Baylor College of Medicine, One Baylor Plaza, T603, Houston, TX 77030 USA

**Keywords:** Exome sequencing, Genome sequencing, Diagnostic utility, Molecular diagnoses, Undiagnosed diseases, Rare disease, Mendelian conditions

## Abstract

The practice of genomic medicine stands to revolutionize our approach to medical care, and to realize this goal will require discovery of the relationship between rare variation at each of the ~ 20,000 protein-coding genes and their consequent impact on individual health and expression of Mendelian disease. The step-wise evolution of broad-based, genome-wide cytogenetic and molecular genomic testing approaches (karyotyping, chromosomal microarray [CMA], exome sequencing [ES]) has driven much of the rare disease discovery to this point, with genome sequencing representing the newest member of this team. Each step has brought increased sensitivity to interrogate individual genomic variation in an unbiased method that does not require clinical prediction of the locus or loci involved. Notably, each step has also brought unique limitations in variant detection, for example, the low sensitivity of ES for detection of triploidy, and of CMA for detection of copy neutral structural variants. The utility of genome sequencing (GS) as a clinical molecular diagnostic test, and the increased sensitivity afforded by addition of long-read sequencing or other -omics technologies such as RNAseq or metabolomics, are not yet fully explored, though recent work supports improved sensitivity of variant detection, at least in a subset of cases. The utility of GS will also rely upon further elucidation of the complexities of genetic and allelic heterogeneity, multilocus rare variation, and the impact of rare and common variation at a locus, as well as advances in functional annotation of identified variants. Much discovery remains to be done before the potential utility of GS is fully appreciated.

## Background

One of the central tenets of genomic medicine has been the idea that undiagnosed Mendelian conditions have a genetic etiology that is both discoverable and can be used to guide development of preventative or therapeutic interventions. Mendelian conditions, while individually rare, altogether impact millions of individuals and families [1, 2], with over 8000 distinct disease traits catalogued to date [3, 4]. Rare single nucleotide variants (SNV), small insertion/deletion (indel) variants, and copy number variants (CNV) have been demonstrated to underlie many Mendelian conditions, leading to the expectation that undiagnosed diseases are largely ‘single-gene’ (monogenic) or ‘single-locus’ disorders [5, 6] that follow classical Mendelian modes of inheritance. The study of Mendelian conditions has had a substantial impact on our understanding of the genomic etiologies and molecular mechanisms underlying rare human disease, and many discoveries have informed mechanistic understanding of more common human conditions as well (reviewed in Posey et al. [7]).

Implicit to the realization of genomic medicine in the clinic is a comprehensive understanding of the relationship between genes and even individual genotypes, and their associated observed clinical phenotypes. Unbiased approaches to interrogation of the genome, such as chromosomal microarray (CMA) and exome sequencing (ES), have driven disease gene discovery. Despite these advances, only 20% (4081/~ 20,000) of identified human protein-coding genes have an established association with one or more disease traits (www.OMIM.org; 19 April 2019). Moreover, the extent to which variation at more than one locus, allelic and locus heterogeneity, and common variants contribute to Mendelian conditions is not yet fully understood, underscoring the notion that disease gene discovery will not be complete with a simple one-to-one cataloguing of genes and disease phenotypes.

Genome sequencing (GS) is the latest broad-based, unbiased testing method to become more readily available, on both research and clinical bases, as next-generation sequencing costs have fallen [8]. Below, we discuss the current landscape of Mendelian disease, the utility of broad-based genomic testing in discovery and diagnostics, and the potential utility of GS in both research and diagnostic settings.

### The current landscape of rare disorders

The progress of Mendelian disease discovery, with 20% of human protein-coding disease genes having been definitively associated with one or more human phenotypes to date, also highlights the tremendous amount of research that remains to be done. Consistent with these data, the pace of novel disease gene discovery does not show evidence of slowing: the US National Human Genome Research Institute (NHGRI)/National Heart, Lung, and Blood Institute (NHLBI)-funded Centers for Mendelian Genomics, which aim to elucidate the molecular etiologies of all Mendelian conditions, report a steady trajectory of 263 novel discoveries per year [7]. Similarly, OMIM has catalogued a steady increase in both the number of phenotypes with an identified genetic etiology, and the number of genes associated with a clinical phenotype [9]. These and other worldwide efforts have elucidated the molecular and genomic architecture of Mendelian conditions, and the broader availability of ES has supported these discoveries.

**Fig. 1:**
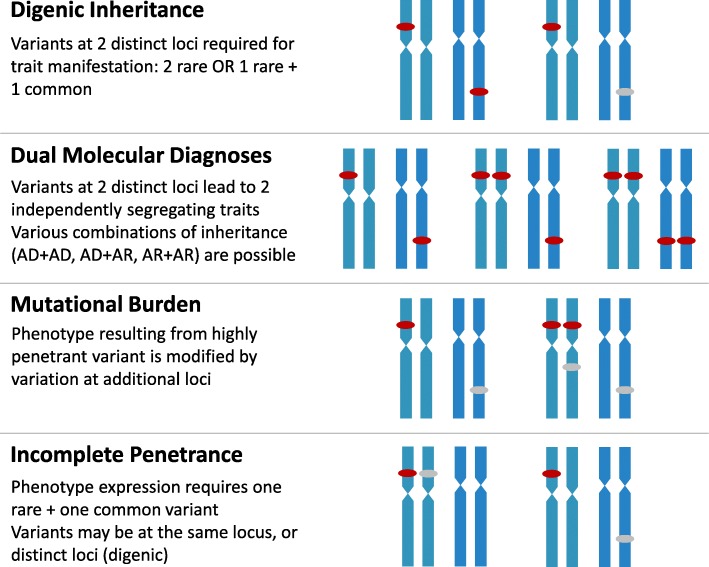
Complex modes of inheritance. Digenic inheritance involves variation at 2 loci that are required for expression of a single Mendelian condition. Most often, both variants are rare, but there have been examples of one rare variant and one common variant at distinct loci leading to expression of a single Mendelian condition. Dual molecular diagnoses occur when an individual has two Mendelian conditions resulting from rare variants at two typically unlinked loci. Mendelian condition pairs can involve one or more modes of inheritance, for example, AD+AD, AD+AR, or AR + AR. Mutational burden is observed when the phenotype associated with a highly penetrant variant is modified by the presence of one or more additional variants which by themselves are not penetrant. Incomplete penetrance can be observed when disease expression requires compound inheritance of one rare and one common variant, either at the same locus, or at unlinked loci. Distinct chromosomes are represented in blue. Rare variants of high penetrance are indicated by red ovals. Common and/or low penetrance variants are indicated by grey ovals. AD – autosomal dominant; AR – autosomal recessive

Mendelian conditions have been associated with a broad range of variant types, including SNVs, indels, CNVs resulting from gains or losses of genetic material that may result in simple duplications or deletions, or more complex genomic rearrangements [10]. Copy neutral genomic structural variants (SVs) and triplet repeat expansions are also etiologic for some Mendelian conditions. The ability to reliably detect many of these variant types through different cytogenetic and molecular genetic technologies has led to the elucidation of Mendelian conditions that, at first glance, do not appear to follow standard Mendelian modes of inheritance. Classically, Mendelian conditions have been categorized as observing autosomal dominant (AD), autosomal recessive (AR), X-linked (XL), or mitochondrial patterns of inheritance. Yet, the study of Mendelian conditions has revealed the extent to which many rare diseases can be characterized by digenic inheritance, dual molecular diagnoses, mutational burden, and compound inheritance of rare and common variants (Fig. 1).

Digenic inheritance, first described in 1994, is defined by the requirement of 2 pathogenic variants at distinct, independently segregating loci, for expression of a single disease condition [11]. Kajiwara et al described 3 families with multiple individuals having retinitis pigmentosa (MIM# 608133), which was known at the time to display locus heterogeneity. They observed that all affected individuals had pathogenic variants in *PRPH2*, but curiously, some unaffected relatives also shared these variants; the risk to offspring of an affected individual was noted to be less than the 50% expected for a dominant Mendelian condition. Only affected individuals had both the variant in *PRPH2* and a second, null allele at an unlinked locus, *ROM1*. More recent discoveries of digenic inheritance include facioscapulohumeral dystrophy type 2 (FSHD2, MIM# 158901), which results from rare variation in *SMCHD1* on chromosome 18 and a permissive *DUX4* allele on chromosome 4 [12]. The *SMCHD1* variant results in relaxation of the chromatin of *DUX4*, similar to the effect of the D4Z4 array contraction in FSHD1 (MIM# 158900), thus leading to a clinically identical dystrophy phenotype [13].

Dual, or multiple, molecular diagnoses (Fig. 1), occur when pathogenic variation at two or more loci leads to expression of two or more Mendelian conditions. Though recognized since the 1960s in individuals who developed hemolytic anemia in combination with thalassemia or sickle cell trait [14, 15], the extent to which such cases occur – and their breadth of molecular diagnoses has only more recently begun to be revealed [16–23]. Pairs of Mendelian conditions can present in an individual as blended phenotypes that may result from overlapping or distinct clinical features, developing contemporaneously or even sequentially over time [16, 24]. The evolution of our understanding of Fitzsimmons syndrome (previously MIM# 270710) illustrates the challenges of relying on clinical ascertainment for such cases [25, 26]. First described in 4 unrelated families as a Mendelian condition involving intellectual disability, spastic paraplegia, short stature, and cone-shaped epiphyses, further study demonstrated that one twin pair diagnosed with Fitzsimmons syndrome had dual molecular diagnoses – trichorhinophalangeal syndrome (MIM# 190350) with a heterozygous variant in *TRPS1* plus Charlevoix-Saguenay type spastic ataxia (MIM# 270550) due to pathogenic variants in *SACS* [21, 27]. A third, unrelated individual with a clinical diagnosis of Fitzsimmons syndrome was found to have a *TBL1XR1* variant responsible for part of the observed phenotype, with no second molecular diagnosis identified. Dual molecular diagnoses are now recognized to account for at least 4% of cases for which molecular testing is diagnostic [16–19, 23], with a diagnostic rate that is even higher (12%) in cohorts of selected phenotypes [22] or in cases with apparent phenotypic expansion (32%) [28]. This frequency is quite likely to increase as more disease genes and genotype-phenotype relationships are discovered.

Multilocus mutational burden (Fig. 1) can impact the expression of disease, both between and within families. Genomic studies of neuropathy support a model whereby an aggregation of rare variants in disease-associated genes can influence clinical severity and can contribute to common complex traits. In an analysis of unrelated families of European descent with peripheral neuropathy, a background mutational load impacting proteins that function in the affected biological network was identified in probands (1.8 additional rare missense variants per individual) compared to controls (1.3, *p* = 0.007) [29]. Only 45% of probands were found to have a highly penetrant, rare variant at a disease gene locus [29]. This analysis was replicated in a distinct Turkish cohort, and zebrafish models demonstrated an epistatic interaction between identified gene pairs [29]. Susceptibility to Parkinson disease can similarly be impacted by a mutational load involving genes that impact lysosomal function [30], and the age of onset of ALS can be modulated by a mutational load in known ALS-associated genes [31]. It is important to note that such multilocus variation may involve variants at one nuclear genome-encoded locus and one mitochondrial genome-encoded locus. For example, nuclear-encoded *TFB1M* has been proposed to influence the hearing loss phenotype associated with MT-*NRN1* (m.1555A > G), which demonstrates intrafamilial phenotypic variation from normal hearing to profound congenital hearing loss [32]. These reports illustrate how mutational burden within a pathway or biological system can modify severity and onset of disease expression.

Incomplete penetrance (Fig. 1) for a Mendelian condition can be a hallmark of more complex molecular pathogenesis. Such conditions can result from a combination of rare and common genetic variants at one or more loci. In the case of nonsyndromic midline craniosynostosis due to pathogenic rare variants in *SMAD6*, low penetrance (< 60%) is observed with *SMAD6* variation alone, but 82% (14/17) of affected individuals had an additional, common *BMP2* allele, demonstrating digenic inheritance of 2 unlinked loci, in this case with one rare variant and one common SNV [33]. Phenotypic expression of *TBX6*-associated congenital scoliosis (TACS, MIM# 122600) similarly requires both a rare loss-of-function (LoF) variant in *TBX6 in trans* with a common, hypomorphic *TBX6* allele; the LoF allele alone is not sufficient for phenotypic expression [34–36]. Lethal pulmonary hypoplasia associated with *TBX4* or *FGF10* also requires compound inheritance of a rare LoF and rare or common hypomorphic allele for expression of disease [37].

**Fig. 2:**
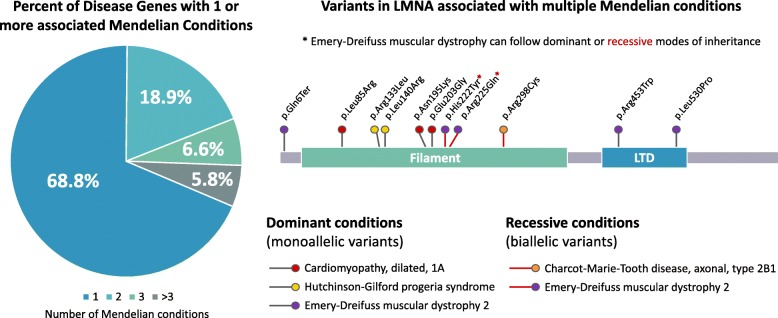
Disease genes can be associated with more than one Mendelian condition. Review of genes associated with disease phenotypes in OMIM (January 2019) reveal that 31% of disease genes have more than one disease phenotype association, with nearly 6% associated with more than 3 Mendelian conditions. Rare variants in LMNA are associated with a variety of both dominantly and recessively inherited phenotypes. LTD - lamin tail domain

Another way in which some Mendelian conditions depart from classical genetic expectations is the occurrence of both dominant and recessive inheritance associated with a single locus, and the observation of more than one Mendelian condition associated with a single locus [38–40]. Indeed, a review of disease-gene relationships in OMIM demonstrates that nearly one-third of genes with an established association with Mendelian disease have been reported in association with 2 or more Mendelian conditions (Fig. 2). Laminopathies, a set of human disease phenotypes resulting from variation in *LMNA*, illustrate this concept well, with diverse disease expression and inheritance patterns including cardiomyopathies (MIM# 115200), neuropathies (CMT2B1, MIM# 605588), skeletal myopathies (Emery Dreifuss muscular dystrophy; MIM# 181350, 616,516), Hutchinson-Gilford progeria (MIM# 176670), and restrictive dermopathy (MIM# 275210). These varied phenotypes result from proposed mechanisms that include differential allelic expression [41], haploinsufficiency associated with late-onset phenotypes [42], dominant negative or GoF associated with early onset phenotypes [42], and digenic inheritance [38, 43, 44].

The complex relationships between Mendelian conditions and their associated genes and genotypes underscore the current challenges of clinical diagnostics and discovery. Inherent to the goal of identifying and characterizing the molecular architecture of Mendelian conditions is ability to detect with sufficient sensitivity and specificity the relevant types of variants. In the next section, we discuss broadly available cytogenetic and molecular genomic assays in the context of Mendelian conditions.

### The advantage of an unbiased assessment

The simple wisdom conveyed by the “streetlight effect” is that by limiting one’s search to the most accessible regions of the genome, one introduces observational bias to a given exploration. In the context of genetic and genomic testing, such bias occurs when one limits discoveries or molecular diagnoses to those which are anticipated. Genome-wide analyses are, by contrast, unbiased in the sense that they do not pre-suppose a particular gene, variant, or locus, as etiologic for a given condition. Karyotyping was first used as a diagnostic tool in 1959, when two clinically recognized conditions were revealed to be caused by chromosomal anomalies: trisomy 21 leading to Down syndrome, and an extra X chromosome leading to Klinefelter syndrome [45, 46]. As techniques to stain the DNA, such as Giemsa-banding (G-banding) were developed, the utility of karyotyping expanded from identification of simple chromosomal anomalies (trisomies, monosomies) to more complex structural rearrangements including deletions, duplications, and translocations, and enabled the field to contextualize these in the setting of several well-characterized clinical phenotypes. Indeed, the unbiased ‘genome-wide’ assessment that karyotyping provided enabled many of these discoveries.

Chromosomal microarray (CMA) techniques brought increased resolution for genome-wide detection of CNVs, and the ability to detect uniparental isodisomy and parental consanguinity. Various studies comparing the diagnostic utility of CMA and karyotyping in pre- and post-natal samples demonstrated an increased diagnostic rate of ~ 6% in postnatal cases, and 2% in prenatal cases [47–49]. One key outcome of these studies was the identification of abnormal findings detected by karyotype, but not by CMA, occurring in 0.9–1.4% of studied cases. A majority of the abnormalities not detected by CMA either exhibited mosaicism, or involved apparently balanced chromosomal rearrangements that would appear copy neutral by array-based technologies. While reciprocal and Robertsonian translocations, which are copy neutral SVs, typically have no direct phenotypic consequence, they increase the risk of unbalanced translocations or chromosomal anomalies in the subsequent generation. In rare cases, they may also lead to disruption of a Mendelian disease gene and consequent disease expression: for example, study of two individuals with clinical diagnoses of Sotos syndrome who were found to have translocations with breakpoints disrupting 5q35 ultimately led to the identification of *NSD1* as the Sotos syndrome gene (MIM# 117550) [50, 51].

Exome sequencing (ES) became the next step in the evolution of genome-wide testing, using next-generation sequencing (NGS) technologies to focus on the coding portions of the genome, in which over 95% of disease-causing variants have been estimated to be located [52]. From both a clinical and research standpoint, the advantage of ES lies in the ability to interrogate almost all ~ 20,000 human protein-coding genes simultaneously for rare SNVs and indels known or suspected to be etiologic for disease. This testing has enabled the identification of dual molecular diagnoses in clinical referral cohorts [16–22], and supports the interrogation of genomic data for multilocus variation impacting phenotypic expression [28–30]. Many groups have analyzed the diagnostic utility of ES in a clinical referral setting, and found that molecular diagnoses are identified in 25–50% of sequential clinical referrals, with a somewhat lower diagnostic rate in cohorts of adult (> 18 years) individuals [17–20, 53, 54]. Objective reanalysis of clinical cases can further increase clinical diagnostic yield [55]. Other groups have compared the diagnostic utility of ES to panel-based testing, essentially comparing analysis of ES data to a ‘virtual gene panel’ designed from masked exome variant data. In a comparison of ES to a 55-gene panel in individuals across all ages with peripheral neuropathy, ES increased diagnostic yield from 22 to 38% [56]. A subsequent study of 145 children with suspected Mendelian disease demonstrated that of 57 cases for which a diagnosis was obtained by ES and for which physicians had recommended gene panel alternatives, nearly one quarter (13/57, 23%) would have remained undiagnosed by any of the proposed alternative gene panels [57]. Despite the demonstrated increase in diagnostic utility for ES, several key challenges remain to improving the sensitivity of ES for detection of etiologic variants: uniformity of sequencing coverage particularly in GC-rich regions, consistent detection and correct annotation of indels [58, 59], and identification of CNVs, particularly small CNVs involving only one or a few exons [60–63]. Notably, an analysis of the diagnostic utility of ES compared to ES + CMA demonstrated a higher diagnostic rate when ES and CMA are performed concurrently, highlighting a continued role for CMA in clinical diagnostics [64].

The utility of these unbiased genome-wide technologies, as tools for both clinical diagnostics and research-based discovery, is clear. While it is intuitive to anticipate that larger NGS studies with greater coverage of the genome will be of greater utility, lessons from karyotyping, CMA, and ES serve as reminders to consider carefully the limitations of each testing method. In the following section, we explore the potential added utility of genome sequencing (GS) in the clinic and the research laboratory.

### The promise of genome sequencing in the clinic

While no longer a new method, GS has fairly recently become more available for clinical diagnostic testing. Analyses of the diagnostic utility of GS have ranged from 21 to 73%, impacted by phenotypes and individual ages studied [65–69]. Comparisons of the diagnostic utilities of GS and ES have been fairly limited to date, but a few groups have shown a modest increase in diagnostic rates of GS; these findings highlight coverage of both coding and non-coding sequences, with typically lower fold-, but more consistent, nucleotide-by-nucleotide coverage of GC-rich regions (including first exons) compared to ES, improved detection of CNVs, and more complete detection of variants associated with common pharmacogenomic alleles. Alfares et al studied 108 individuals for whom array comparative genomic hybridization (aCGH) and ES were non-diagnostic, and identified 7 cases for which GS identified a molecular diagnosis: these cases included a *PHOX2B* repeat expansion, a large deletion encompassing *TPM3*, and a deep intronic variant in *TSC2*, as well as 3 individuals with a missense variant in *ADAT3* and 1 individual with a missense variant in *SLC35A2* that were simply not detected by the initial ES (though the authors noted that BAMs were not available for re-analysis of ES data in these 4 cases) [70]. An additional 3 molecular diagnoses (all coding variants) not detected on initial ES, were identified by GS and subsequent ES reanalysis. Some have also considered the potential utility of GS as a screening, rather than diagnostic, study. In an analysis of molecular findings of screening GS in a cohort of apparently healthy adults, 22% (11/50) were identified to have a previously unknown disease risk, 100% (50/50) were found to be a carrier for an AR Mendelian condition, 96% (48/50) were identified as having a pharmacogenomic variant impacting drug metabolism, and between 6 and 40% of individuals were identified as being in the top 10th centile of risk by polygenic risk score analysis for 8 cardiometabolic conditions [71].

Another potential advantage of GS is the ability to interrogate rare variants encoded by the mitochondrial genome. While some groups have taken advantage of off-target reads from ES and other capture-enriched NGS datasets to identify mitochondrial genome-encoded variants, [72, 73] the presence of a high fraction of nuclear mitochondrial DNA segments (NUMTs) in the nuclear genome, coupled with the relatively low read depth coverage of the mitochondrial genome using these approaches can confound variant calling, particularly for heteroplasmic variants. The application of a single pair of back-to-back primers to amplify the mitochondrial genome can be used to eliminate NUMT contamination and achieve high-coverage mitochondrial genome sequence [74, 75]. In the clinical setting, such testing could be ordered concurrently with ES or GS, or as part of a step-wise diagnostic approach – this requires a priori diagnostic suspicion of a mitochondrial condition. Mitochondrial genome-encoded variants may also be identified from GS data, and this has recently been illustrated by the identification of a rare variant in MT-*ND4* (m.11778G > A) conferring a diagnosis of Leber hereditary optic neuropathy (MIM# 535000) [76], and the identification of a rare homoplasmic variant in MT-*TI* (m.4300A > G) conferring a diagnosis of primary familial hypertrophic cardiomyopathy [77]. Methods development to detect lower frequency heteroplasmic mitochondrial variants from GS datasets is underway [78], suggesting that GS may become a viable option for interrogation of both nuclear and mitochondrial genomes with high sensitivity and specificity in the near future.

One weakness of the lower-fold coverage of GS is the reduced sensitivity to detect and correctly identify mosaic variants, particularly those of low allele fraction [79]. The power to detect mosaic variants is influenced by the allele fraction of the variant and the depth of coverage, with lower allele fraction variants requiring a high depth of coverage. Studies modeling this relationship between allele fraction and read depth have indicated that the detection of somatic mosaicism as low as 5% at 95% sensitivity requires a read depth of at least 140-fold, which is relatively cost-prohibitive in the context of GS [80]. One approach to address the potential for parental germline mosaicism for identified, apparently de novo variants from trio-GS data is the application of high read depth NGS to further interrogate genomic positions of interest [81].

In clinical practice, diagnostic reporting of ES and GS findings focus primarily on established disease genes, and variants that are known or strongly suspected to be pathogenic based on objective evidence [82]. Improved functional annotation of noncoding variants identified by GS will be necessary to resolve those that are truly pathogenic from those that are benign, and this represents a key step in increasing the diagnostic yield and clinical utility of GS. Despite the potential opportunity for GS-based diagnostic testing, complete realization of its diagnostic utility in the clinic awaits further discovery in the field of Mendelian disease and additional advances in computational and technological approaches to genomic analyses.

### Exploring the potential of genome sequencing through research

Genome sequencing in the research setting offers the opportunity to explore the full contribution of non-coding variants -- including SNV, CNV, and copy neutral structural variants (SV) -- to Mendelian disease. Certainly, many examples of non-coding variation contributing to Mendelian disease have been described, such as the *ELP1* (formerly *IKBKAP*) variant that affects splicing observed in individuals of Ashkenazi descent with familial dysautonomia (MIM# 223900) [83, 84], low frequency regulatory SNVs in *RBM8A in trans* with a 1q21.1 deletion in individuals with thrombocytopenia-absent radius syndrome (TAR, MIM# 274000) [85], or the polymorphic poly-thymidine tract in intron 9 of *CFTR* that can impact expression of cystic fibrosis (MIM# 219700) in the presence of the p.Arg117His *CFTR* variant *in cis* [86–88]. Noncoding SVs affecting regulatory regions have also been associated with Mendelian disease, with several examples of loci for which distinct SVs produce very distinct phenotypes [6, 89]. For example, *SHH* has been observed in association with (1) holoprosencephaly and cleidocranial dysplasia in a woman with a de novo 6;7 reciprocal translocation with one breakpoint 15 kb upstream of *SHH* [90], and (2) pre-axial polydactyly-hypertrichosis in a family found to have a 2 kb deletion upstream of the *SHH* promoter [91]. These reports illustrate the complexity of genotype-phenotype relationships observed with noncoding SNVs and SVs, and highlight the tremendous potential for discovery of novel molecular mechanisms afforded by GS.

To comprehensively address genotype-phenotype relationships involving noncoding variants, the field will need to improve upon current methods for interpretation of the functional and regulatory effects of novel noncoding SNVs and SVs. This will almost certainly require a multi-pronged approach, with efforts aimed at improved computational tools for predicting functional effects of noncoding variants [92–94], development of in vitro or cell-based functional assays applicable to gene regulation or protein function, and concomitant analysis with other broad-based ‘-omics’ approaches such as RNAseq and metabolomics. Several recent studies have demonstrated the potential for success with these methods. Gasperini et al recently reported the large-scale perturbation of 5920 candidate gene enhancer elements, and used single-cell transcriptome data to determine the effects on nearby gene expression; this approach yielded 664 potential *cis* enhancer-gene pairs [95]. Others have used RNAseq to search for aberrant splicing or expression levels attributable to noncoding variants identified by GS. This has worked particularly well for identifying variants with tissue-specific effects in muscle and mitochondrial phenotypes [96, 97]. Analysis of de novo variants from trio-GS (proband + parents) data is yet another approach to identify putative pathogenic noncoding variants in individuals with apparently sporadic disease [98], and a deep-sequencing approach can enable detection of low-level parental germline mosaicism, which can impact recurrence risks within a family and may be undetected by GS and/or targeted dideoxy Sanger sequencing of parental DNA [99]. Though many efforts to address the role of non-coding variation in disease have focused on identifying etiologic rare variants, the relationship between combinations of rare and common variants at one or more loci in disease is also not yet fully explored [34–37].

Expansion of GS techniques to include long-read sequencing enables genome assembly with greater access to complex regions of the genome and improved mapping to the human genome reference sequence. Long-read sequencing supports identification of SVs, particularly copy neutral changes not identified by CMA or short-read sequencing approaches; this approach was recently applied to 15 individual genomes across multiple ethnicities to identify and sequence resolve over 99,000 SVs [100–103]. Long-read GS also supports phasing of variants over longer genomic segments [100–102]. These advantages have been balanced by 2 key tradeoffs: (1) increased sequencing costs which can range from $750–1000/Gb for long read technologies, compared to $7–250/Gb for short read technology; and (2) the potential for increased sequencing error rates which can range from < 1 to 13% for long read technologies, compared to 0.1–1.0% for short read technologies [104]. Recent work has demonstrated a move toward significantly lower error rates and improved cost-effectiveness with long-read sequencing [105, 106]. The potential diagnostic efficacy of SV detection by long-read GS is supported by a recent report of an individual diagnosed with Carney complex due to a ~ 2 kb deletion involving exon 1 of *PRKAR1A*, a CNV not detected using short-read genome sequencing [107]. Interrogation of complex regions of the genome, such as HLA typing for transplant candidates, and loci with known pseudogenes, are additional potential applications for long-read technologies [108, 109].

As GS is increasingly used in the clinical and diagnostic settings, the field will need to consider how best to weigh factors such as cost, error rates, sequencing breadth and depth of coverage, and molecular diagnostic utility in determining whether ES, GS, GS combined with other -omics, or even reanalysis of existing variant data are most appropriate for a given case or cohort.

## Conclusions

As with each of the genome-wide, unbiased cytogenetic and molecular techniques that have been developed, GS offers the potential for further growth of clinical molecular diagnostics, driven by new discovery of genes and molecular mechanisms associated with Mendelian disease. More work is needed to develop methods to support prioritization and functional classification of variants identified by GS, particularly non-coding and copy neutral structural variants, as well as methods to fully interrogate trinucleotide repeats and more complex, repetitive and/or GC-rich regions of the genome before the utility of GS is fully realized.

## Data Availability

All data presented are published and/or publicly available.

## Abbreviations

aCGH: Array comparative genomic hybridization
AD: Autosomal dominant
AR: Autosomal recessive
CMA: Chromosomal microarray
CNV: Copy number variant
ES: Exome sequencing
GS: Genome sequencing
Indel: insertion/deletion variant
SNV: Single nucleotide variant
SV: Structural variant
XL: X-linked
